# Multi-Analytical Framework to Assess the In Vitro Swallowability of Solid Oral Dosage Forms Targeting Patient Acceptability and Adherence

**DOI:** 10.3390/pharmaceutics13030411

**Published:** 2021-03-19

**Authors:** Abdul Latif Ershad, Ali Rajabi-Siahboomi, Shahrzad Missaghi, Daniel Kirby, Afzal Rahman Mohammed

**Affiliations:** 1 Aston Pharmacy School, College of Health and Life Sciences, Aston University, Birmingham B4 7ET, UK; ershada@aston.ac.uk (A.L.E.); D.J.KIRBY1@aston.ac.uk (D.K.); 2 Colorcon Inc., Harleysville, PA 19438, USA; asiahboomi@colorcon.com (A.R.-S.); SMissaghi@colorcon.com (S.M.)

**Keywords:** film coating, solid oral dosage forms, in vitro swallowability, dysphagia, adherence, acceptability, swallowability index

## Abstract

A lack of effective intervention in addressing patient non-adherence and the acceptability of solid oral dosage forms combined with the clinical consequences of swallowing problems in an ageing world population highlight the need for developing methods to study the swallowability of tablets. Due to the absence of suitable techniques, this study developed various in vitro analytical tools to assess physical properties governing the swallowing process of tablets by mimicking static and dynamic stages of time-independent oral transitioning events. Non-anatomical models with oral mucosa-mimicking surfaces were developed to assess the swallowability of tablets; an SLA 3D printed in vitro oral apparatus derived the coefficient of sliding friction and a friction sledge for a modified tensometer measured the shear adhesion profile. Film coat hydration and in vitro wettability was evaluated using a high-speed recording camera that provided quantitative measurements of micro-thickness changes, simulating static in vivo tablet–mucosa oral processing stages with artificial saliva. In order to ascertain the discriminatory power and validate the multianalytical framework, a range of commonly available tablet coating solutions and new compositions developed in our lab were comparatively evaluated according to a quantitative swallowability index that describes the mathematical relationship between the critical physical forces governing swallowability. This study showed that the absence of a film coat significantly impeded the ease of tablet gliding properties and formed chalky residues caused by immediate tablet surface erosion. Novel gelatin- and λ-carrageenan-based film coats exhibited an enhanced lubricity, lesser resistance to tangential motion, and reduced stickiness than polyvinyl alcohol (PVA)–PEG graft copolymer, hydroxypropyl methylcellulose (HPMC), and PVA-coated tablets; however, Opadry^®^ EZ possessed the lowest friction–adhesion profile at 1.53 a.u., with the lowest work of adhesion profile at 1.28 J/mm^2^. For the first time, the in vitro analytical framework in this study provides a fast, cost-effective, and repeatable swallowability ranking method to screen the in vitro swallowability of solid oral medicines in an effort to aid formulators and the pharmaceutical industry to develop easy-to-swallow formulations.

## 1. Introduction

The reliance on solid oral medications for effective pharmacotherapy requires patients to possess an inherent ability and willingness to swallow medication. Swallowing problems are among one of the most prevalent issues significantly afflicting the elderly and pediatric populations [1]. Dysphagia is the medical term for physiological difficulty in swallowing and can be broadly sub-classified as oropharyngeal and esophageal types [2]. Conservative estimations suggest that 16–22% of the world’s population and more specifically up to 40% of the general US population experience a range of tablet swallowing difficulties, highlighting a widespread concern [3,4]. Notably, up to 68% of elderly residents in US care homes are identified as dysphagic and reported to be receiving polypharmacy [5,6]. Furthermore, the incidence of presbyphagia (characteristic age-related swallowing difficulty) is predicted to be as high as 70% globally due to an ageing world demographic [7].

Currently, research efforts to improve the physical swallowability of solid oral dosage forms (SODFs) are primarily addressing alternative dosage forms. However, the lack of standardization, drug loading limitations, taste–odor masking issues, cost-intensive means of manufacturing, and difficulty in characterization have rendered alternative oral dosage forms inadequate or with limited success. It is pertinent to note that not all drugs can be manufactured via alternative manufacturing methods such as freeze-drying, sublimation, wet granulation, pelletization, or casting technology. Moreover, concerns regarding patient familiarity and failing to address oral acceptability factors such as after-taste, grittiness, post-swallow residue, smoothness, and stickiness remain outstanding with regard to mini-tabs, multi-particulates, 3D printed tablets, oral films, orally disintegrating tablets, buccal tablets, reconstitution powders/granules, effervescent tablets, chewable tablets, scored tablets for splitting, and freeze-dried wafers [8,9,10].

Since tablets are the most common dosage form available on the market, as well as the existence of an ageing world population susceptible to geriatric syndromes such as swallowing difficulties, paramount investigation is warranted to improve the swallowing experience of oral medications [8]. Understanding the medication-taking behavior of patients and knowing which product attributes drive patient acceptance are keys to the successful development and marketing of new medicines, as well as helping to ensure adherence [9]. Results of a systematic review and an investigation into the solid oral medication characteristics that influenced adherence and acceptability found that memorability, swallowability, and palatability were key considerations that dictated patient medication intake behavior [11,12]. Recent publications from the FDA on guidelines for the design of SODFs cited swallowing difficulties as one of the major causes of non-adherence [4]. In addition, the FDA stated that the lack of a film coat reduces the tablet mobility during swallowing, hence advocating the inclusion of film coating in the design of SODFs. The absence of a film coat on tablets has been shown make them more difficult to swallow than their coated counterparts via in vivo clinical scintigraphy, X-ray fluoroscopy, and patient questionnaire studies [13,14,15,16]. Common film coatings in the market are predominantly low-molecular-weight polyvinyl alcohol or hydroxypropyl methylcellulose (hypromellose) polymers that impart a smoothness and enhanced visual appeal in comparison to relatively rough uncoated tablets, resulting in an improved swallowing experience [17,18]. Furthermore, the use of water-soluble polymers has found commercial success and practical application in medication lubricants (e.g., Gloup^®^ and PillGlide^®^), as well as in a range of modified foods for dysphagic patients that are carrageenan-, gelatin-, and xanthan gum-based formulations [19,20,21]. Hofmanová et al., (2019) conducted a randomized double-blind study on 84 adults to rank their preference of different placebo-coated tablets with respect to the ease of swallowing [22]. The study found that the presence of a film coat improved the swallowability of tablets; tablets coated with Opadry^®^ EZ (a hypromellose guar gum combination) was identified to possess the most desirable tablet profile with respect to slipperiness, mouthfeel, and palatability.

During the physical transport of medicines in the mouth, two opposing physico-mechanical forces occur at the tablet–mucosa interface, resulting in friction. While the gripping motion is an essential feature of the tongue to propel boluses towards the pharynx, the resilience of ingestible material (e.g., a tablet) affects the amount of work that mouthparts must do during oral transportation, which can affect the sensory perceptions of slipperiness, stickiness, and smoothness [23,24,25]. Qazi and Standing (2017) provided an overview of in vitro models mimicking swallowing phases, but the majority of methods related to the esophagus or mastication process [26]. The intricate anatomy, complex coordination of physiology, and biomechanics of the swallowing process reflect the limited existing designs of in vitro oropharyngeal swallowing models [26]. To date, there is no consensus or harmonious definition of acceptance criteria for in vitro evaluative techniques to screen or adequately test for the swallowability or the palatability of tablets [27,28]. This is further compounded by the problem of limited understanding and guidance concerning pharmaceutical sensory analysis despite advanced recognition and implementation within the food sector [29,30]. Due to the outstanding need for easy to replicate quantitative measurements of in vitro swallowability, this study, for the first time, aimed to develop suite of in vitro analytical tools designed to assess critical properties, namely friction–adhesion, viscosity, and surface tension, as an effective screening method to predict the swallowability of various film-coated tablets [22,30,31,32,33,34,35]. The multianalytical framework is underpinned by a swallowability by design (SbD) paradigm that serves as an effective formulation screening method to predict the swallowability of various film-coated tablets. The SbD integrates the current understanding of pharmaceutical oral acceptability and food science of palatability that entails the assessment of critical properties governing the swallowability of oral dosage forms, i.e., friction–adhesion, viscosity, and surface tension. To aid the study design, a range of film coating compositions (commercial and new compositions) were studied to establish the discernibility of the analytical methods with the ultimate objective of formulating an indicative swallowability index parameter.

## 2. Materials and Methods

### 2.1. Materials

Sodium polyacrylate was purchased from Magnacol Ltd. (Newtown, Wales). Magnesium stearate, parafilm, and double-sided polytetrafluoroethylene (PTFE) adhesive were purchased from Fischer Scientific (Loughborough, UK). The polyvinyl alcohol–polyethylene glycol graft copolymer (Kollicoat^®^ IR, denoted as KIR) was provided by BASF GmbH (Ludwigshafen, Germany). Albumin, α-lactose monohydrate, gelatin type A from porcine (bloom strength 90), λ-carrageenan from red seaweed (denoted as λ-C), hydroxypropyl methylcellulose (HPMC; grade 2910 and average M_w_ of 8000 Da), and polyvinyl alcohol (referred to as PVA; average M_w_ 31,000 of Da and 98–99% hydrolyzed) were all purchased from Sigma-Aldrich, (Poole, UK). Potassium phosphate, sodium fluoride, and calcium chloride were purchased from RM Marketing (Essex, UK). Aerosil^®^ 200 was from Evonik GmbH, (Wesseling, Germany), and Avicel^®^ PH102 was from DuPont (Delaware, USA). Propyl-methacrylate-polyvinyl acetate phthalate (PMPP) clear resin was obtained from Formlabs GmbH, Germany. Opadry^®^ EZ (referred to as OEZ) formulations were obtained from Colorcon (Colorcon Ltd., Dartford, UK).

### 2.2. Methods

#### 2.2.1. Preparation of Tablet

The required quantities of lactose (79% *w/w*) and Avicel^®^ PH102 (19% *w/w*) were blended for 10 min using Erweka AR 403 all-purpose equipment in an acrylic glass cubical blender (mixing angle of 20° at 150 rpm) (Erweka^®^ GmbH, Germany). This was followed by the addition of Aerosil^®^ 200 (1% *w/w*) and then magnesium stearate (1% *w/w*) individually for a minute each to achieve a uniform powder blend. A Specac Ltd. bench-top semi-automatic hydraulic press with a flat-faced, 13 mm, stainless steel evacuable die tooling set (Slough, UK) was used to compress 500 mg tablets with 150 MPa of force.

#### 2.2.2. Preparation and Characterization of Artificial Saliva

A mix of 0.1% albumin, 0.5% sodium carboxymethylcellulose, 0.062% potassium phosphate, 0.01% sodium fluoride, and 0.07% *w*/*v* calcium chloride were dissolved in ultrapure water [36]. The pH was evaluated using a pH meter (Model 320, Mettler-Toledo, Greifensee, Switzerland); the artificial saliva’s pH was 7.05 ± 0.04. A viscosity of 5.39 ± 0.15 cPs at 25 °C was measured by an automated microviscometer (*n* = 5) using a 1.8 mm capillary tube with a 1.5 mm steel ball of 7.7 g/cm^3^ density and an inclination angle of 80° that was calibrated with the viscosity standard N26 (with a standard viscosity of 4.2 ± 0.2 mPa.s) (Anton Paar Ltd., St Albans, UK).

#### 2.2.3. Film Coating Apparatus

Tablets were coated in a fluidized bed coater to a 3% theoretical weight gain at an air flow fluidization rate of 16 m/s using a mechanical agitator to oscillate the spray chamber at a 3 mm amplitude at 16 Hz. Tablets were pre-heated at 60 °C for 10 min using a 303 stainless steel 1/8′′ JJAU-SS atomizing spray nozzle (Spraying Systems Co., Wheaton, IL, USA) and Caleva Mini Coater Drier II (Caleva Process Solutions Ltd., Dorset, UK).

#### 2.2.4. Coating Solution Preparations

Kollicoat^®^ IR, Opadry^®^ EZ, hydroxypropyl methylcellulose (E5) and polyvinyl alcohol were prepared as 10% *w*/*v* coatings solutions using an overhead digital mixer with cold ultrapure water (100 rpm, room temperature). All coating solutions shown in Table 1 contained 10% *w*/*w* Kollicoat^®^ IR. Binary coating solutions (Table 1) included the addition of gelatin or λ-carrageenan at solid levels of 1 or 3% *w/w*. Ternary compositions consisted Kollicoat^®^ IR (10% *w/w*), with gelatin fixed at 3% *w/w* with the additions of λ-carrageenan at 1 or 3% *w/w*. The resulting aqueous coating solutions were passed through a Silverson^®^ L5MA high shear mixer (Silverson Machines Ltd., Waterside, UK) at 2000 rpm for 10 s to evenly disperse and solubilize the remaining undissolved polymers within the coating solution. A Fisherbrand^TM^ FB15051 ultrasonicator was used to degas the coating solutions for 5 min at room temperature. All coating solutions prepared in this study possessed a viscosity below 500 cP, as determined with a Brookfield DV-11 +Pro viscometer using spindle 4.

#### 2.2.5. Multi-Analytical Framework to Assess In Vitro Swallowability

##### Change in Hydrated Film Coat Layer Thickness

A Canon^®^ T7i 800D DSLR camera with an electro-focus 18–55 mm and macro 100 mm lens was used to microscopically examine the dimensional changes of liquid uptake occurring within film coats (*n* = 6). Tablets were immersed in artificial saliva heated to 37 ± 2 °C within a petri dish positioned under an overhead fluorescent light source and over a hotplate. Equations (1) and (2) were used to calculate the change in the radial swelling fronts of the film-coated tablets (up to 60 s). The projected area distribution of hydrated films was calculated using the ImageJ software (an open source image processing tool) with a graduated scale bar from the high-speed recordings still-shots. Each gel-forming stage (swelling front, diffusion front, and erosion front) was calculated at the onset point of tablet core disintegration using the 3D viewer plugin and volume area calculation on ImageJ.

(1) $$Change\;In\;Gel\;Layer\;Thickness=\;\frac{\left(T_{n}-T_{0}\right)}{T_{n}}\;\times \;100$$

(2) $$Cumulative\;Change=\;\sum^{\;}\frac{\left(T_{1}-T_{0}\right)}{T_{1}}+\frac{\left(T_{2}-T_{0}\right)}{T_{2}}+\;\ldots +\frac{\left(T_{n}-T_{0}\right)}{T_{0}}\times 100$$

where *T_n_* is the thickness at time interval ‘*n*’, *T_0_* is the initial thickness, and *T*_1_ is the first thickness measurement

##### In Vitro Wettability

The ImageJ contact angle measurement tool was used to calculate the immediate droplet spherical cap area and tablet contact angles at 10 and 20 s to determine the in vitro wettability of the film-coated tablet [37]. The imaging setup consisted of a Komodo camera swivel tripod affixed with a precision spirit level, and an additional incandescent 10-watt light source was used for brightness control. A drop volume of 10 μL from a micropipette was used for the sessile drop technique to negate an increased apparent wetting effect caused by gravity. Digital images obtained from high speed video recordings were analyzed by drawing a baseline tangent using DiameterJ (a nanofiber diameter characterization tool) and Brugnara plugin (drop shape analysis tool) connecting both the right and left tri-phase points, and a computer estimation for the droplet circularity was used to calculate the formed wetting angle. The length of each center-line was averaged, and an axial thinning algorithm was applied to prevent underestimations of the wetting angle from a 2D image [38]. The contact angle measurement was repeated six times.

##### In Vitro Coefficient of Sliding Friction

**Figure 1:**
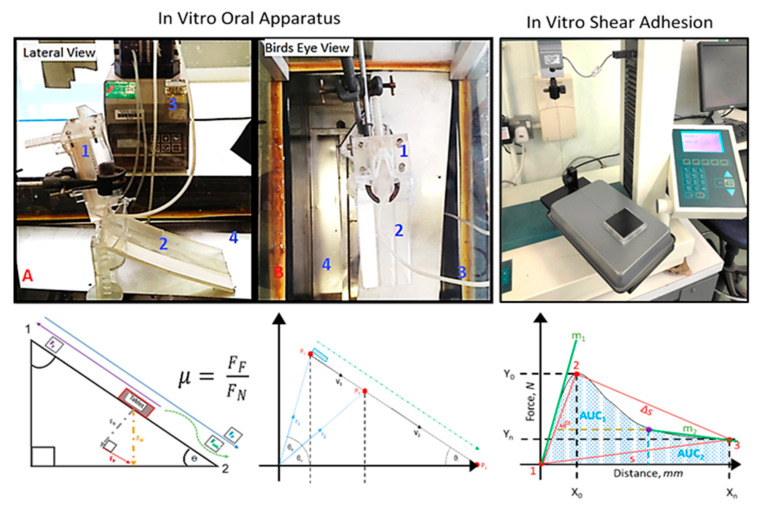
Illustration of basic friction–adhesion model using the in vitro oral apparatus (**left**, **A**—lateral view, **B**—birds eye view) and in vitro shear adhesion device (**right**) and respective free body diagrams used to determine critical swallowability factor’s coefficient of sliding friction and work of adhesion. The in vitro oral apparatus required tablets to be introduced via an entry slot unwetted (1), a gated mechanism separated the tablet from the wetted ramp (2) maintained by a peristaltic pump (3), and a plexiglass environmental chamber (4) mounted onto a steam bath maintained the internal conditions, i.e., temperature (37 ± 2 °C) and humidity (65% relative humidity). The in vitro shear adhesion device used a 3DP tablet holder to affix 9 tablets on the underside to be dragged at a defined speed across a mucosa-mimicking surface (sodium polyacrylate or polytetrafluoroethylene (PTFE)). A frictionless and 10 N load cell was used to improve recording accuracy.

A Formlabs Form 2 High Resolution Stereolithographic 3-D printer and Tinkercad^®^ graphic design software (Autodesk^®^, San Rafael, CA, USA) was used to model and produce a computer-aided designed incline ramp with an adjustable height frame to manipulate the inclination angle (see Figure 1). The in vitro oral apparatus was designed as a non-anatomical oral mucosa-mimicking closed system. A stereolightography 3D printed (SLA 3DP) incline ramp was fabricated to sit beneath a plexiglass entry ramp to introduce tablets; the apparatus was enclosed within a plexiglass casing that was fitted onto a steam bath to enable the control of the temperature and humidity; the time taken to complete the total course (20 cm ramp length) was used to determine the relative dynamic coefficient of friction with respect to the clear resin PMPP polymer surface using Coulomb’s friction model (see Equation (3)) [39]. Tablets were placed within a gated vertical ramp designed with 0.5 mm perforated pores that allowed for a constant stream of artificial saliva (at an optimal flow rate of 1 mL/s) to wet the surface of the in vitro oral apparatus (see Figure 1). The vertical ramp was designed to introduce the tablet into the table-top designed ramp at a defined inclination angle of 45° that mimicked the pharyngeal bolus position [40,41]. The transition time taken for a tablet to complete the main ramp course was used to calculate the acceleration caused by gravity in order to deduce the net force occurring along the ramp. A series of trigonometric calculations involving the natural tendency of the tablet mass to slide at a specified angle of inclination determined the normal perpendicular force, as well as the sliding friction force (Equations (A1)–(A8); see Appendix A). The normal force (*F_N_*) and frictional force (*F_F_*) was used to determine the coefficient of sliding friction (*CoF*) of the film-coated tablets (*n* = 6), as illustrated in Figure 1.

(3) $$F_{F}=\mu F_{N}$$

##### In Vitro Shear Adhesion

A modified stainless steel FT200 coefficient of friction device fixture with a carbide-coated frictionless pulley for a Hounsfield tensometer (Model H10KS, Tinius Olsen Ltd. Surrey, UK) was developed and used to ascertain force–distance time plots across oral mucosa-mimicking surfaces (see Figure 1). An HTE QMat Professional TestZone version 3.1 S series software program was used to acquire data from S/T/L 10 N series Z-beam sensor. A friction method in accordance with the American Society for Testing and Materials international standard ASTM D1894 (test method for static and kinetic coefficients of friction) was developed with a 0.5 N normal force of the weighted 3D printed tablet holder. A stereolithographic 3D printed sledge was designed to firmly hold 9 tablets on its underside and was pulled across an ultrapure-water-saturated cast of sodium polyacrylate or a double-sided PTFE rubber-based tape (10 cm in length). A test speed of 300 mm/min was used for an extension range of 50 mm, with an approach speed of 0.005 mm/min post-optimization of experimental variables (*n* = 6).

Subsequent derivations from the force–distance plots generated by Hounsfield tensometer software include the maximum detachment force plus the work of relative adhesion (W_a_) to comprehend the in vitro wet-slip profile and stickiness of tablets. Riemann’s definite integral sum and a trapezoidal approximation rule was used to determine the W_a_ of the shear adhesion curves with the assumptions that the force equation f(x) is a scalar valued function of single variable distance (Equation (4)). A Lagrange interpolation partition method and mid-point rule were used to determine the linear polynomial of the curve (see Figure 1 and Equation (5)). The static friction coefficient was calculated as the ratio between the force required to initiate tablet movement and the normal force of the tablet holder mass, while the dynamic friction coefficient was the ratio between the average force during tablet movement and the normal force. The integration of the area under the curve yielded the relative work of adhesion (see Figure 1).

(4) $$Area\;of\;Trapezium\;\left(A\right)=\;\frac{1}{2}h\left(b_{1}+b_{2}\right)$$

where *h* represents the perpendicular height and *b*_1_ and *b*_2_ are the parallel base sides of the trapezium; a Lagrange interpolation goes through the designated points within the region of interest that is determined graphically from a polynomial equation obeying linearity. Following the trapezoidal rule for approximating the area under the curve within a specified region of interest, Δ*x* is deduced as $\frac{b-a}{n}$ and $x_{i}$ represents the arbitrary interpolation coefficients and are prefixed functions determined from a+iΔx across the x-axis (specified partition subdivisions).

(5) $$\overset{b}{\underset{a}{\int }}f\left(x\right)dx\;\approx \;\frac{\Delta x}{2}\;f\left(x_{a}\right)+2f\left(x_{1}\right)+\ldots +2f\left(x_{n-1}\right)+f\left(x_{n}\right)$$

Reimann’s definition for the true area under the curve given by the integral is approximated by Δ*x* representing the perpendicular height of the trapezium, where *b*_1_ and *b*_2_ ≈ (*a*, *b*) are the definite integral limits of *f*(*x*), also denoted [*a*, *f*(*a*)] and (*b*).

### 2.3. Statistical Analysis

Statistical analyses were conducted using an ANOVA (GraphPad Prism 7.2). Results are expressed as means ± standard deviation (std). The statistical significance threshold was set at *p* < 0.05.

## 3. Results and Discussion

The primary aim of this work was to develop a suite of techniques capable of discerning appreciable micro-metric changes on film-coated tablet surfaces. The multianalytical framework in this study was modelled as a laminar segmental system depicting time-dependent and -independent stages during the swallowing process due to the complexity of such. Changes in hydrated film thickness studies coupled with the evaluation of surface wetting and subsequent changes were studied to understand the tablet surface during the stationary phase in the oral cavity. Tablet mobility was evaluated by studying the shear adhesion measurements and coefficient of friction to fully appreciate the consequences of the changes that occurred during the stationary phase. In order for these tests to be fully discriminating, a range of different coating compositions was evaluated, including commercially available compositions and new binary and ternary systems. The typical swallowing procedure for tablets deviates from normal oral processing experienced with food, whereby the bulk substrate is ingested wholly without mastication or communition but is triturated and wetted with an aqueous layer of saliva and water whilst moving along mucosa. An effective degree of wetting is required to form a coherent, soft viscous mass that does not result in adhesive interactions preventing displacement from oral mucosa [42]. The measurement of the apparent contact angle and change in hydrated film thickness could allow for insight into the tablet–mucosa interaction during stationary periods of the transitioning behavior of tablets during swallowing with small volumes of physiological fluids, such as saliva in the oropharyngeal tract.

**Figure 2:**
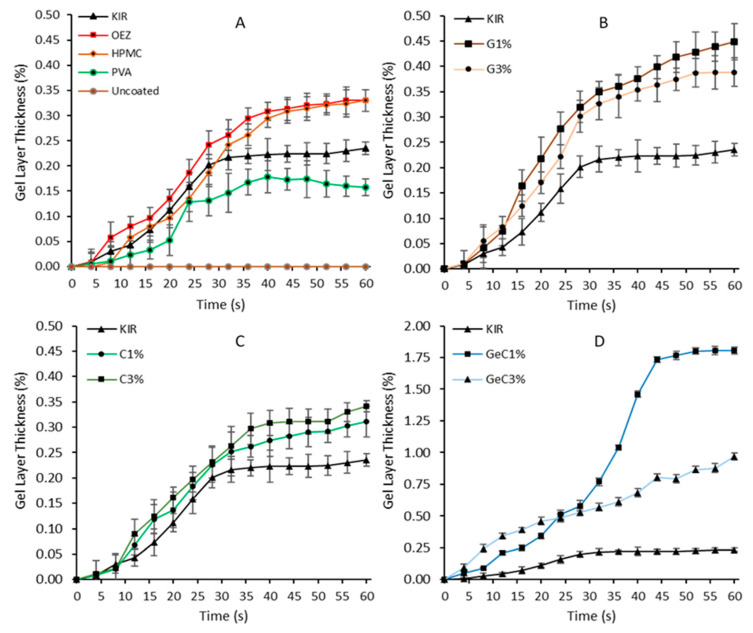
Quantitative analysis of the axial gel layer thickness over time of film-coated tablets, as calculated using a high-speed recording camera with macro lens and imaging software for 60 at 4 s intervals. Uncoated tablets commenced disintegration and did not swell when immersed in water. Graph (**A**) represents commercial grade film coats, gelatin formulations are shown in graph (**B**), λ-carrageenan shown in graph (**C**), gelatin: λ-carrageenan combination formulations are represented in graph (**D**).

Figure 2 shows that uncoated tablets did not present any observable change when in contact with the artificial saliva; hence, a change in thickness could not be established. KIR film-coated tablets did not inherently possess a pronounced swelling profile; ascertaining quantitative measurements of KIR swelling fronts proved difficult due to its highly soluble nature, as seen in Figure 2, and was also confirmed by the high-speed recording camera that showed that KIR film-coated tablets solubilized quickly and peeled off after 40 s. Film coats with the lowest artificial saliva uptake were the most soluble and retained the poor hydration characteristic of KIR [43]. Figure 2 shows that PVA-based film-coated tablets possessed the lowest change in film thickness, followed by Kollicoat^®^ IR, HPMC, and Opadry^®^ EZ (Figure 2). At 12 s of immersion time, Opadry^®^ EZ imbibed greater amounts of artificial saliva at 0.08% than the PVA film-coated tablets at 0.03% (ANOVA, *p* < 0.001). After 24 s of immersion, the PVA film coats plateaued at a 0.12% change in thickness, whereas both the HPMC- and OEZ-based film systems reached a plateau at 40 s at a higher change in thickness of 0.28% (ANOVA, *p* < 0.05).

**Figure 3:**
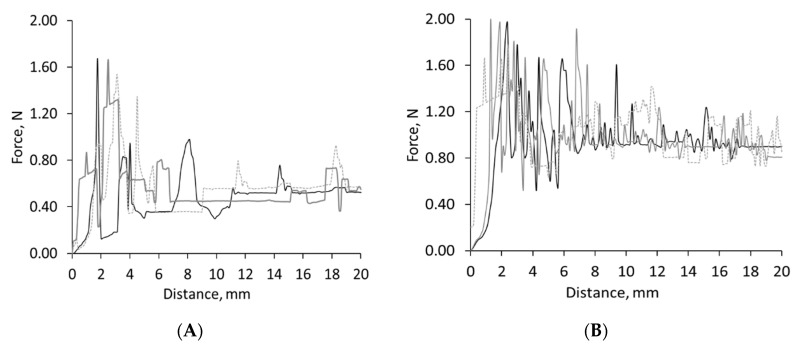
In vitro shear adhesion profile plotted as a function of distance for uncoated tablets. The oral gliding performance of tablets can be determined using a modified Hounsfield tensometer friction sledge across two non-anatomical oral mucosa-mimicking surfaces wetted with artificial saliva (polytetrafluoroethylene as graph (**A**) and sodium polyacrylate as graph (**B**)). Uncoated tablets displayed an erratic creep profile caused by a stick–slip motion resulting in friction re-strengthening and the fragmentation of the tablet, as depicted by the frequent large stress drops of the shearing peaks in both oral mucosa-mimicking surfaces.

Interesting trends were observed with the addition of gelatin and/or λ-carrageenan swelling agents in KIR film systems. The addition of gelatin at 3% *w/w* moderately improved the water-imbibing behavior of KIR, whilst binary λ-carrageenan (denoted as λ-C) formulations at 3% *w/w* similarly showed a 0.089% increase at 12 s (Figure 2). However, the greatest increase in the change in hydrated film thickness was observed with ternary formulations containing gelatin and λ-carrageenan. These differences in hydration behavior could be described by understanding the molecular structure and potential synergistic activity that may have emerged due to the competing forces within the film coat. The special triple helical structure of gelatin possesses a range of electrostatic, hydrophilic–hydrophobic, and hydrogen bonding interactions. The latent high-water absorption capacity of gelatin is explained by the diffusion of water molecules localized by the activation of hydroxy alkoxy functional groups via hydrogen bonding and neighboring hydrophobic amino groups within the triple helix strands [44,45]. λ-C is devoid of the relatively hydrophobic 3,6-anhydro-α-D-galactopyranose and consequently incapable of forming pockets of imbibed water due to hydrophilic–hydrophobic contortions, and, being highly ester sulphated, it is thus easily solubilized by the numerous hydrogen-bond-forming oxygen atoms. Furthermore, the ester sulphate distribution of λ-C is randomly distributed; the presence of three ester sulphates per λ-C monomer averts extensive gelation via the prevention of aggregation in comparison to kappa or iota carrageenan and in place promotes viscous solutions, resulting in limited liquid imbibition [46]. Ternary compositions of gelatin with λ-C showed improved water-imbibing behavior compared to KIR in isolation (ANOVA, *p* < 0.05). The combination of gelatin and λ-carrageenan at 3% *w/w* required 20% less time than gelatin-based film coats to significantly exceed 1.50% change in hydrated film thickness at 40 s, as seen in Figure 3. λ-C contains sulphated functional groups that promote physical constraints that affect its absorption capacity. However, when λ-C is combined with gelatin, linear strands of λ-C are potentially capable of bedding themselves within its triple helix strands. Consequently, extensive peptide carbonyl group hydrogen bonding occurs within gelatin’s cylindrical grooves, resulting in gelatin to fill with water and leading to faster hydration [47,48]. The addition of small amounts of swellable polymers within KIR networks has demonstrated their ability to temporarily entrain water physically held by capillary forces, hydrogen bonding, and coil–helix complexation [49]. Moreover, studies have demonstrated the physical compatibility of binary compositions consisting of ethylcellulose aqueous dispersion (Aquacoat ECD) with either a polyvinyl alcohol–polyethylene glycol copolymer or propylene glycol with polysaccharide gums alginate, carrageenan, and/or guar; the multi-polymer blends have been found to exhibit improved mechanical and functional properties [50,51,52].

Upon placing a tablet within the mouth for ingestion, the behavior at the effective contact area against the mucous membrane influences the adhesion behavior for a short period [53]. An effective degree of wetting is required to form a coherent, soft viscous mass that does not result in adhesive interactions that prevent displacement from oral mucosa [54]. In order to further understand the changes in hydrated film thickness, contact angle measurements were carried out to ascertain the ease of spreadability of artificial saliva. The evidence from the contact angle study showed that the inclusion of hydrophilic polymers capable of imbibing water, such as gelatin and λ-carrageenan up to 5% *w/w*, within films can significantly improve the wetting behavior of the surface coat. Table 2 shows the novel formulations possessed contact angles below 90°, such measurements are a uniform signature of a hydrophilic wetting profile. In this study, it was found that the uncoated tablets generally rapidly took up water within 10 s and commenced disintegration due to the highly water-soluble nature of the filler; therefore, contact angles of the tablet core could not be accurately obtained [55].

The rank order of lowest immediate apparent contact angles for commercial film-forming polymers were OEZ < HPMC < PVA < KIR at 91.6°, 94.3°, 96.2°, and 97.2°, respectively (Table 2). However, after 20 s of wetting time, the rank order of lowest apparent contact angles was OEZ < KIR < HPMC < PVA at 81.3°, 83.2°, 86.1°, and 88.6°, respectively (Table 2). The greatest surface spreading and depth of droplet immersion was observed after 10 s for KIR, possibly due to the presence of the more hydrophilic PEG groups that were also responsible for its high solubility (40% *w*/*v* solutions could be prepared) and tendency to swell [56,57,58]. From Table 2, it can be seen a significant linear correlation could be observed between the contact angle and wetting time (see Appendix B, Table A1 for a list of the Spearman’s rank correlation coefficients for contact angle study). A clear difference of contact angle with respect to time was observed between KIR and binary film coat compositions, as the inclusion of increasing concentration of λ-C demonstrated significant decreases of wetting angles in comparison to KIR (ANOVA, *p* < 0.05). Interestingly, despite the immediate contact angle and subsequent measurement at 10 s, no gelatin film coat formulation surpassed an angle of 90°, unlike λ-C (ANOVA, *p* < 0.05) (Table 2). The contact angle of λ-C film-coated tablets, dropped below 80˚ after 20 s, thus highlighting λ-C’s greater affinity for water molecules. At a wetting time of 20 s, an increase in λ-C concentration from 1 to 3% *w/w* solid inclusion significantly decreased the apparent contact angle from 75.8° to 71.0° (ANOVA, *p* < 0.05). Compared to KIR, after 20 s, the apparent contact angle was reduced by 13% to 84.3° (ANOVA, *p* < 0.05). The lower contact angles exhibited by OEZ, HPMC, gelatin, and λ-C could possibly be attributed to hydrogen bond acceptor counts; while λ-C has 20, OEZ possesses 16 due the presence of guar gum, leading to more polarized surfaces upon hydration than gelatin [59,60]. Gelatin film-coated tablets displayed a more hydrophobic wetting response in comparison to λ-C film-coated tablets regardless of the of solid content (ANOVA, *p* < 0.05). (Table 2). It was hypothesized that gelatin predominately exhibits a droplet immersion behavior, whereas λ-carrageenan and guar (within OEZ) adopt a radial wetting pattern allowing for greater spreading, as shown in Table 2 [61,62].

**Figure A1:**
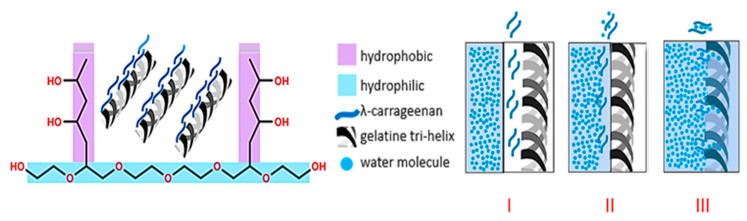
Schematic illustration of Kollicoat^®^ IR: gelatin:λ-carrageenan ternary network water imbibition theory exhibiting favorable surface coat properties for low friction, low adhesion, and soft texture formation. Phase I hypothesizes that the linear carrageenan chains coat the gelatin triple helix because gelatin possesses numerous relative hydrophobic amino segments that pair with carrageenan’s backbone. The blending of Kollicoat is believed to induce the gelatin: λ-carrageenan complex to associate with polyvinyl alcohol chains that are less hydrophilic than polyethylene glycol. Phase II shows that during water contact, carrageenan molecules readily bond with water molecules, thus resulting in phase III where intricate inter- and intra-hydrogen bonding creates a passage into gelatin’s triple helix that promotes extensive synergistic swelling.

In contrast, the ternary mixture of gelatin and λ-C at 1% *w*/*w* possessed a contact angle of 93.2° at 0 s (ANOVA, *p* < 0.05), whereas increasing ternary component λ-C to 3% *w/w* inclusion further reduced the wetting angle to 89.6° (ANOVA, *p* < 0.05). Regardless of the formulation, after a wetting time of 20 s, gelatin: λ-C at 3% *w/w* inclusion displayed the lowest wetting angle at 71.1°, 15% less than KIR (ANOVA, *p* < 0.05). The current findings showed that augmenting gelatin-coated tablets with λ-C-coated demonstrated superior swelling capacities than can be attributed to the likely synergism accounting for low hydrophilic wetting angles and superior hydration capacity (Appendix C; Figure A1 in the appendix illustrates the proposed physical mechanism of λ-C and gelatin synergism). Ternary combinations of λ-C and gelatin exhibit favorable wetting and hydration synergism at low concentration incorporations. The addition of water-soluble polymers within film coating compositions has been shown to effectively improve the wettability of tablet surfaces and to assist in the formation of a hydrated film layer that could potentially exhibit hydrodynamic lubrication during the mobile phases of tablet–mucosa contacting surfaces.

**Figure 4:**
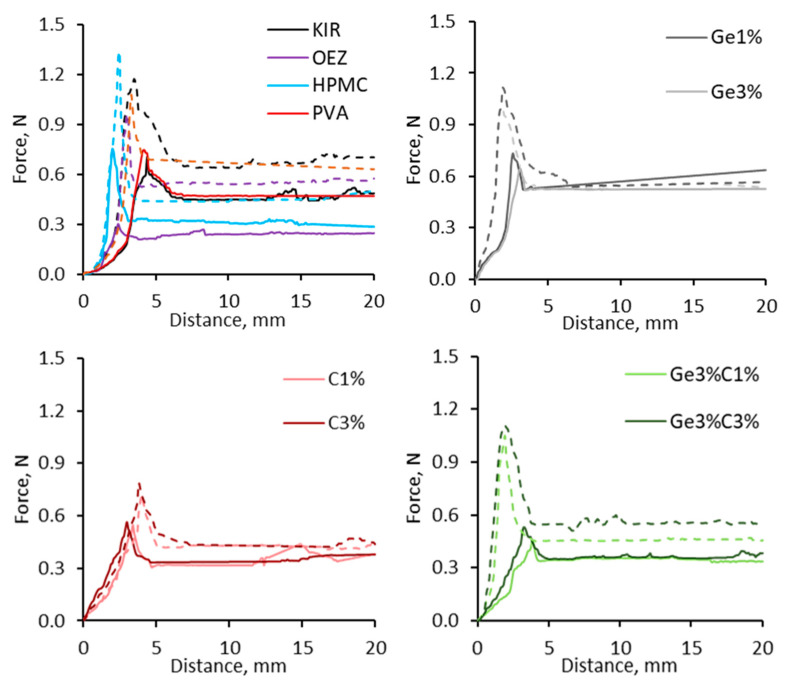
Graphs to show the in vitro oral gliding performance and shear adhesion profile of various film-coated tablets using a modified Hounsfield tensometer across an in vitro, non-anatomical model using artificial saliva wetted sodium polyacrylate (dashed lined) and polytetrafluoroethylene ramp surface (solid lines). HPMC and PVA possessed higher initiating detachment forces and displayed a greater gliding resistance in comparison to Opadry^®^ EZ (OEZ), which possessed the most optimal slippery profile in comparison to all film-coated formulations (ANOVA, *p* < 0.05). The addition of water-soluble polymers within KIR (Kollicoat^®^ IR) imparted lubricating properties, as substantially lower peak forces and gliding forces were recorded.

To study the critical forces occurring during the dynamic phases of swallowing acting between tablet–mucosa interface, the adhesion and coefficient of friction were studied using a modified tensometer and a novel in vitro oral apparatus set up, respectively. For the tensometer-based experiments (see Figure 1), two different oral mucosa-mimicking surfaces—sodium polyacrylate and polytetrafluoroethylene ramp—were studied to explore the influence of hydrophilic and hydrophobic contact surfaces, respectively. Initial studies focused on studying the shear adhesion profile of uncoated tablets. Figure 3 and Figure 4 represent the force–distance graphs of uncoated tablets and various film-coated tablets (both commercial and novel compositions), respectively. Two distinguishable regions could be evaluated from the non-linear force–distance response; the initial spike is representative of the peak displacement force required to detach the adhering film-coated tablet from the ramp surface, thus representing the ease of oral clearance during static interactions between the tablet and mucosa. A subsequent stress drop region devolves into a linear variation of shear stress, which is indicative of the wet-slip profile (minimal energy to induce and maintain tangential motion).

Uncoated tablets in this study displayed a highly adhesive behavior, particularly on the sodium polyacrylate substrate (Figure 3); the high shearing strengths recorded for uncoated tablets could be attributed to the rougher surface of uncoated tablets in comparison to the smoother finished film-coated tablets. It is likely the water migration within uncoated tablets induces a strongly absorbed surface layer, causing the fragmenting behavior of the boundary layer and resulting in successive high stresses during tangential motion. The calculation of the initial peak area determines the work of relative adhesion, the energy required to separate adjacent surfaces that is akin to the yield point of the hydrated film. Uncoated tablets required the highest work of adhesion of 8.95 J/mm^2^ on the sodium polyacrylate mucosa-mimicking surface. This trend was also observed on the PTFE substrate; however, substantially less adhesion energy was required to initiate the movement of uncoated tablets at 1.99 J/mm^2^—a reduction of 77.65%, as seen in Figure 4 (ANOVA, *p* < 0.0001). Figure 4 shows that Opadry^®^ EZ required the least amount of work (Wa) for strain-induced displacement at 1.28 J/mm^2^ on the PTFE ramp, whereas ternary system gelatin 3% *w/w*: λC 1% *w/w* needed 42% more force at 2.19 J/mm^2^ when using the PTFE substrate. Kollicoat^®^ IR and HPMC required the most energy to induce motion, as their respective work of adhesion profiles were 3.72 and 3.52 J/mm^2^, respectively, when using the hydrophobic PTFE ramp surface (ANOVA, *p* < 0.05); meanwhile, on the hydrophilic sodium polyacrylate surface, Kollicoat^®^ IR’s and HPMC’s work of adhesion values were 4.06 and 4.25 J/mm^2^, respectively (ANOVA, *p* < 0.05). The addition of gelatin and/or λ-C into KIR coating systems resulted in both the detachment and dynamic force to reduce and did not exceed 0.5 N (ANOVA, *p* < 0.05 compared to Kollicoat^®^ IR). From Figure 4, it can be seen the maximum limiting resistance force gradually decreased, possibly due to the mobilization of the film coat; however, differences in the hydrophilicity and texture of PTFE tape and sodium polyacrylate ramp substrate surfaces increased the apparent dynamic slip force profile of all film coat systems.

Sodium polyacrylate creates a water-locked upper surface; the presence of hydrogen-bonding moieties within a film coat matrix creates a strong affinity for an aqueous layer [63]. This results in low interfacial energies, facilitating wetting and inter-diffusion, increasing the contact time and thus forming semi-permanent adhesive bonds [64]. The presence of artificial saliva here continuously bathed the tablets; wetting of the film coat thus promoted adhesion. High normal load pressures led to the suppression of sliding, whereas low pressures promoted frictional slip behavior; noticeably, Opadry^®^ EZ most effectively imparted lubricity in comparison to other formulations, as observed in Figure 4. The literature has demonstrated that polysaccharide gums such as xanthan, carrageenan, guar, and konjac gum possess unique abilities to dissipate frictional energies through their saccharide side branch structures due to the greater extent of coil–helix transformation that imbibes superior volumes of water, creating thin aqueous sheets for improved gliding [65,66,67]. Furthermore, the results confirmed that the critical shear force needed to initiate lateral motion of binary film-coated tablets in comparison to uncoated, KIR, HPMC, or PVA-coated tablets was greater than the minimum transitional force of the common, aforementioned commercial coating systems, an indication of the least resistant wet slip behavior. As the wetted sodium polyacrylate surface formed an adhesion zone with the film-coated layer, the artificial saliva present at the interface was subsequently adsorbed by the presence of hydrophilic polymers within the film coat. Upon the swelling and hydration of the film layer, the resulting film microgel expanded, facilitating the polymer chains to interpenetrate the macromolecular network of sodium polyacrylate [21]. In comparison to the hydrophobic PTFE ramp surface, non-bonding physical interactions such as repulsion appeared to be the determining factors for film coat adhesion. Unlike the upper bound water layer observed on the sodium polyacrylate surface, residual water was freely available across the PTFE tape. PTFE is sheathed with layers of highly electronegative fluorine atoms, and the presence of poorly polarizable carbon–fluorine bonds results in repulsive surfaces when in contact with aqueous media; PTFE exhibits exceptionally low friction values similar to that of mucosa [61]. PTFE resists wetting—as tablets are dragged across the hydrophobic surface of PTFE, a thin waterbed film formed and simultaneously absorbed by the film coat layer due to the presence of polarizable hydroxyl groups to allow for transitioning across the PTFE surface.

**Figure 5:**
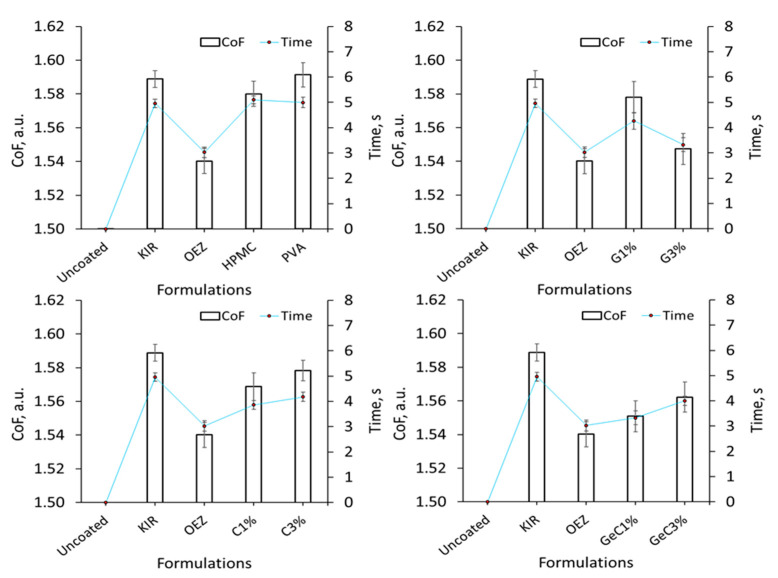
Determination of the sliding coefficient of friction (CoF) and mobility time of film-coated tablets with KIR as the control and fixed at 10% *w*/*w*. Coulomb’s friction model *F_F_* = µ*F_N_* was used to determine the CoF by using the weight to run its course on a 3D SLA printed in vitro oral apparatus, and it can be seen that the binary and/or ternary addition of λ-carrageenan and/or gelatin in combination effectively reduced the in vitro friction forces exhibited by the control KIR. OEZ yielded the lowest resistance to tangential motion, exhibiting a relatively superior wet-slip profile than any film coating system (ANOVA, *p* < 0.05).

Similarly, the evaluation of the CoF of film-coated tablets using our in house in vitro oral apparatus setup were comparable to the in vitro shear adhesion data. Uncoated tablets strongly adhered to the 3D printed oral ramp surface as rapid disintegration occurred, thus forming a chalky residue; consequently, the coefficient of sliding friction could not be ascertained. Figure 5 also shows that Kollicoat^®^ IR and PVA possessed similar CoF values, whereas HPMC possessed a moderately lower CoF than both commonplace film formers. The rank order of lowest coefficient of sliding friction values were OEZ at 1.53 a.u. followed by combination of gelatin 3% *w/w* and λ-C 1% *w/w* at 1.54 a.u., both of which exhibited significantly less resistance to gliding than Kollicoat^®^ IR at 1.59 a.u. (ANOVA, *p* < 0.05). A similar trend was observed in the in vitro oral apparatus setup, whereby OEZ possessed the most superior gliding ability in comparison to commonly employed film coating compositions PVA, HPMC, and Kollicoat^®^ IR (Figure 5). Increasing the concentration of gelatin and λ-C led to a moderately prolonged mobility time during the in vitro coefficient of sliding studies, indicating that such polymers in isolation exhibited a concentration-dependent tendency to resist motion (Figure 5). From Figure 5, it can be seen that the binary gelatin film coat systems possessed CoF values of 1.58–1.54 a.u. from 1% to 3% *w/w* inclusion. Binary film coat compositions displayed contrasting behaviors, as λ-C at 1% *w/w* and gelatin at 3% *w/w* exhibited the greatest shear thinning properties; however, increasing the concentrations of λ-C within ternary gelatin:λ-C from 1% to 3% *w/w* inclusion systems appeared to form an increasingly elastic flow corresponding to the moderate rise in CoF values from 1.54 to 1.57 a.u.

The inclusion of water-soluble polymers improved the wetting profile by enabling the formation of peripheral water-entrained sheets on the film surface, thus reflecting the lower wetting angles critical for the founding of a thinly hydrated film layer. KIR possesses relatively low viscosity and low molecular weight, and it is not a highly branched co-polymer, which consequently makes hold less water to dissolve and thus reveal the rougher tablet core surface; this was reflected by KIR’s high friction–adhesion profile, which was indicative of lower water-binding properties in comparison to Opadry^®^ EZ and novel gelatin and/or λ-C film compositions, whose higher water-imbibing capabilities lent lubricating properties (Figure 5). As water content is an integral feature of polymeric materials’ ability to flow, to be easily wetted and increase the resident water capacity within coats were demonstrated to impart a desirable slippery texture in this study.

While sensory tests in the format of questionnaires, surveys, and observations are utilized to comprehend patient perception of swallowability and investigatory in vivo instrumentation (mainly in the form of video-based fluoroscopy or manometry and, less commonly, gamma scintigraphy) aims to understand the physiological process of swallowing, there remains an outstanding need for a comprehensive quantitative in vitro mathematical model that harmonizes critical factors governing the swallowability of a solid bolus [13,14,15]. Data from the measurements presented in this study, including contact angle, hydration thickness, work of adhesion, and coefficient of friction, were triangulated to understand the interplay between different measurements and develop a quantitative metric to study swallowability. The swallowability index proposed in this study is a quantitative indicator that describes the mathematical relationship between the lubricating factor of hydrated film thickness with respect to the combined forces of friction–adhesion and wettability. The determination of the change in hydrated film thickness, wettability, coefficient of friction, peak detachment force, and work of adhesion provide time-independent physiological inferences of the oral events occurring between the tablet surface and oral cavity. The total oral transit time for swallowing oral medications is specific to each individual patient and is thus a subjective process whereby each individual can require variable amounts of time to ingest. The calculation of the swallowability index (see Equation (6)) with respect to distinct time-specific change in hydrated film thickness from 4 to 20 s can provide potential insight into the zonal performance of film-coated tablets within the oral cavity. It is pertinent to note that the in vitro swallowability data comprise an indicative scale for in vitro preformulation purposes. Table 3 and Table 4 detail color-coded swallowability matrixes that rank all formulations based on sequential coating recipe complexity and swallowability performance (Table 5), respectively.

(6) $$\mathrm{Swallowability}\;\mathrm{index}\;=\;\frac{1000GL}{CA\;\times \;W_{a}\;\times \;10F\;\times \;CoF}$$

where *GL* is the axial gel layer thickness (%), *CA* is the contact angle, *W_a_* is the work of adhesion, *F* is the force required to initiate movement, and *CoF* is the coefficient of friction.

## 4. Conclusions

A “swallowability by design” concept necessitates the optimization of four crucial factors that dictate the gliding performance and influence palatability of swallowing solid oral medication; the work of adhesion, coefficient of friction, viscosity (soft solid rheology), and wettability (surface tension). This study showed that the multianalytical framework can quantitatively measure the physical properties governing time in-/dependent in vivo oral swallowing events. Furthermore, novel binary/ternary gelatin and λ-carrageenan film-coated tablets formed a slippery wet mass that possessed hydrophilic wetting regimes that did not impede tablet mobility, which could help ensure safer and easier oropharyngeal ingestion. For the first time, this study presents a swallowability index model that can effectively discriminate and distinguish the in-vitro performance of various film-coated tablets of increasing compositional complexity against oral mucosa-mimicking surfaces. The suite of analytical techniques in this study has demonstrated potential to be a cost-effective and robust in vitro modelling system that can aid formulators across the pharmaceutical industry to develop easy-to-swallow tablets to help improve patient adherence and acceptability by examining the surface properties governing the physical factors governing swallowing.

## Figures and Tables

**Table 1:** Composition of control, binary and ternary coating solutions consisting main film forming polymer Kollicoat IR prepared as a 10% *w/w*-based solution with secondary component gelatin type A or λ-carrageenan at either 1 or 3% *w/w* inclusion.

Formulation	Kollicoat^®^ IR, % *w/w*	λ-Carrageenan, % *w/w*	Gelatin Type A, % *w/w*
Control	10	0	0
			
Binary Composition	10	1	0
	10	3	0
	10	0	1
	10	0	3
			
Ternary Composition	10	1	3
	10	3	3

**Table 2:** Apparent contact angle measurements with respect to time using sessile drop technique of film-coated tablets. Values equal to and below 90° correspond to increasingly hydrophilic surfaces in contrast to values above 90 corresponding to relatively more hydrophobic surfaces (un-wetting systems). Measurements were taken in triplicate and reported as mean value ± standard deviation. * The immediate apparent contact angle was recorded as the instant stable droplet contact on the tablet surface. HPMC: hydroxypropyl methylcellulose; PVA: polyvinyl alcohol.

Formulation	Immediate *		10 s		20 s	
	Mean	Std	Mean	Std	Mean	Std
Uncoated	0	0	0	0	0	0
Kollicoat^®^ IR	97.17	1.46	89.92	1.59	83.32	1.33
Opadry^®^ EZ	91.64	1.83	87.43	1.34	81.03	1.37
PVA	96.24	1.46	90.53	1.49	88.62	1.23
HPMC	94.28	1.21	89.24	1.36	86.13	1.16
C 1% *w/w*	93.06	1.92	88.73	1.21	75.77	1.31
C 3% *w/w*	88.67	1.24	81.97	1.25	73.03	1.83
Ge 1% *w/w*	98.37	1.37	93.07	1.15	80.87	1.31
Ge 3% *w/w*	104.17	1.55	91.17	1.04	82.87	1.82
Ge 3%: C1% *w/w*	93.23	1.53	86.07	1.54	76.33	1.22
Ge 3%: C3% *w/w*	89.57	1.64	81.87	1.42	71.08	1.36

**Table 3:** Swallowability matrix of coated tablet formulations of sequential increasing complexity, as determined by the swallowability index ranked from poor (red), pass (orange), good (yellow), very good (lime green), to excellent (dark green).

In-Vitro Oral Transit Time, s					
	4	8	12	16	20
Unc.	0.00	0.00	0.00	0.00	0.00
PVA	0.11	0.17	0.37	0.54	0.82
HPMC	0.23	0.76	1.05	1.35	1.86
KIR	0.14	0.61	0.82	1.10	1.83
OEZ	1.19	4.82	5.42	6.78	7.99
C1%	0.51	1.19	3.46	6.20	6.64
C3%	0.60	1.40	4.49	6.18	7.41
Ge1%	0.36	1.56	2.61	3.97	6.10
Ge3%	0.29	1.41	2.22	3.19	4.36
Ge3%C1%	1.46	2.78	5.49	6.77	7.87
Ge3%C3%	1.42	2.34	5.65	6.93	9.47

**Table 4:** Swallowability matrix of coated tablet formulations of sequential presentational performance, as determined by the swallowability index ranked from poor (red), pass (orange), good (yellow), very good (lime green), to excellent (dark green).

In-Vitro Oral Transit Time, s					
	**4**	8	12	16	20
Unc.	0.00	0.00	0.00	0.00	0.00
PVA	0.11	0.17	0.37	0.54	0.82
KIR	0.14	0.61	0.82	1.10	1.83
HPMC	0.23	0.76	1.05	1.35	1.86
Ge3%	0.29	1.41	2.22	3.19	4.36
Ge1%	0.36	1.56	2.61	3.97	6.10
C1%	0.51	1.19	3.46	6.20	6.64
C3%	0.60	1.40	4.49	6.18	7.41
Ge3%C1%	1.46	2.78	5.49	6.77	7.87
Ge3%C3%	1.42	2.34	5.65	6.93	9.47
OEZ	1.19	4.82	5.42	6.78	7.99

**Table 5:** Colour-coded matrix to rank the in-vitro swallowability of film coated tablets using the swallowability index derived from the triangulation of critical physical factors governing the swallowing process.

Swallowability Index	Swallowability Rank	Colour Map
0	Poor	
0 < SI < 1	Pass	
1 < SI < 2	Good	
2 < SI < 4	Very Good	
≥ 4	Excellent	

## Data Availability

The data presented in this study is contained within this article.

## Appendix A

Step 1—Solving for *F_F_*

(A1) $$F_{Net.}=F_{P}-F_{F}$$

(A2) $$F_{Net.}^{T}=m\cdot A$$

(A3) $$m=\;\frac{w}{g_{n}}$$

(A4) $$A=\;\frac{l}{t^{2}}$$

(A5) $$F_{P}=F_{W}\cdot sin\theta$$

(A6) $$F_{W}=m\cdot g_{n}$$

(A7) $$F_{F}=\;F_{P}-F_{Net.}^{T}$$

Step 2—Calculating *F_N_*

(A8) $$F_{N}=F_{W}\cdot cos\theta$$

Step 3—Calculating the Dynamic *CoF*

(A9) $$\mu =\;\frac{F_{F}}{F_{N}}$$

where *F* is the force, *F_F_* is the frictional force, *F_N_* is normal force, *μ* is the coefficient of friction (*CoF*), *F_Net._* is the net force, *F_P_* is the natural force (sliding tendency), *m* is the mass, *A* is the acceleration, *w* is the weight (in kilograms), *g_n_* is the specific gravity of earth constant, *t* is the time, *F_W_* is the work done, and *Ɵ* is the inclination angle.

## Appendix B

**Table A1:** Contact angle with respect to the wetting time of film-coated tablets (immediate, 10-, and 20-s time intervals).

Formulation	*r* ^2^
Uncoated	N/A
Kollicoat^®^ IR	0.99993
Opadry^®^ EZ	0.97955
PVA	0.92344
HPMC	0.98178
C 1% *w*/*w*	0.92883
C 3% *w/w*	0.99932
Ge 1% *w/w*	0.99507
Ge 3% *w/w*	0.99842
Ge 3%: C1% *w/w*	0.99383
Ge 3%: C3% *w/w*	0.99089
